# Antibody-Dependent Enhancement in Flavivirus Infections: From Fc Receptor Signaling to Vaccine and Therapeutic Design

**DOI:** 10.3390/v18080916

**Published:** 2026-08-20

**Authors:** Yiling Li, Zonghui Wu, Yingchao Cha, Wei Pang, Le Sun

**Affiliations:** 1 Yunnan Provincial Key Laboratory of Public Health and Biosafety, Department of Pathogen Biology and Immunology, School of Basic Medicine, Kunming Medical University, Kunming 650500, China; bell06@163.com (Y.L.); wu061655@outlook.com (Z.W.); 15126583419@163.com (Y.C.); 2 Yunnan Provencial Key Laboratory of Cross-Border Infectious Diseases Control & Drug Innovation, Department of Pathogen Biology and Immunology, School of Basic Medicine, Kunming Medical University, Kunming 650500, China

**Keywords:** flavivirus, antibody-dependent enhancement (ADE), Fc receptor

## Abstract

Infections caused by flaviviruses, including dengue virus (DENV), Zika virus (ZIKV), and West Nile virus (WNV), can lead to severe hemorrhagic or neurological disease. Antibody-dependent enhancement (ADE) remains a major obstacle to the development of safe flavivirus vaccines and antibody-based therapeutics. ADE includes ADE of infection, in which antibodies increase in viral entry, replication, or infection load, and ADE of disease, in which antibody-dependent inflammatory and immunopathological responses exacerbate disease severity. Previous reviews have largely addressed the general virological and immunological mechanisms of ADE, but few have integrated antibody isotypes and subclasses, Fc-region modifications, Fc receptor diversity, host FcγR polymorphisms, and complement activation within a translational framework. Consequently, how these factors jointly shape ADE of infection, progression to ADE of disease, and individual risk remains incompletely understood. Here, we synthesize evidence linking antibody properties, Fc receptor expression and signaling, FcγR genetic variation, and complement regulation to both forms of ADE. We then discuss how these findings can inform antigen selection, Fc engineering, complement-informed intervention, and systems serology-based risk stratification. By connecting mechanistic evidence with vaccine and therapeutic development, this review offers a framework for designing safer flavivirus interventions and advancing individualized assessment of ADE risk.

## 1. Introduction

The Flavivirus genus comprises enveloped, positive-sense single-stranded RNA viruses with a genome of approximately 10–11 kb. The genome encodes three structural proteins (capsid (C), pre-membrane (prM) and envelope (E)) and seven non-structural proteins (NS1, NS2A, NS2B, NS3, NS4A, NS4B and NS5) [1,2]. These viral proteins coordinately mediate viral replication and host immune damage. While most flavivirus infections manifest as self-limiting febrile illnesses, severe cases can be life-threatening. Based on tissue tropism and clinical presentation, pathogenic flaviviruses are primarily classified into two categories: viscerotropic viruses, such as dengue virus (DENV) and yellow fever virus (YFV), which can induce severe vascular endothelial dysfunction leading to hemorrhagic fever, vascular leakage and shock, and neurotropic viruses, including West Nile virus (WNV) and Zika virus (ZIKV), which frequently invade the peripheral and central nervous systems to cause severe neurological diseases such as encephalitis, meningitis or Guillain–Barré syndrome [1,2,3].

Flaviviruses are primarily transmitted by hematophagous arthropods, with humans serving as either amplifying hosts (e.g., for DENV and ZIKV) or incidental dead-end hosts (e.g., for WNV) [4]. The geographical expansion of vectors and increased vector–human contact have elevated the risk of flavivirus infection. These viruses cause hundreds of millions of infections worldwide annually, representing a major public health burden globally [5]. Furthermore, diseases caused by mosquito-borne flaviviruses are growing in both frequency and diversity. Consequently, there is an urgent need for effective vaccines and specific antiviral treatments against flaviviruses. However, the development of flavivirus vaccines faces numerous challenges. For instance, the FDA-approved quadrivalent dengue vaccine CYD-TDV (Dengvaxia^®^) confers limited protective efficacy and is associated with a significantly increased risk of severe dengue in certain populations [6]. Similar challenges have been encountered in the development of ZIKV and WNV vaccines [7].

Antibody-dependent enhancement (ADE) is a broad phenomenon in which pre-existing antibodies increase viral infection and/or worsen disease. In this review, ADE of infection refers to antibody-associated increases in viral entry, replication, or infection burden, usually through Fc receptor- or complement receptor-mediated uptake of virus–antibody complexes. ADE of disease refers to antibody-associated worsening of clinical or pathological outcomes and may involve increased viral burden, Fc-mediated inflammatory signaling, vascular dysfunction, and tissue injury. Evidence of ADE of infection in vitro does not, by itself, establish ADE of disease in vivo [8,9].

Although the mechanism underlying ADE has not been fully elucidated, ADE of infection is commonly explained by the antibody-dependent increase in pathogen load (ADIPL) model. According to this model, virus particles opsonized by non-neutralizing or sub-neutralizing antibodies form immune complexes that engage Fcγ receptors (FcγRs) on susceptible cells, including monocytes and macrophages. This process promotes viral internalization and cellular infection and may increase the systemic viral burden. Progression from ADE of infection to ADE of disease, however, additionally involves Fc receptor-mediated signaling and downstream immunopathological responses, including altered cytokine production, inflammation, vascular dysfunction, and tissue injury [7]. Thus, increased infection and enhanced disease are related but biologically and evidentially distinct outcomes. The magnitude and consequences of both forms of enhancement may be modulated by intrinsic antibody properties, including isotype, subclass, and post-translational modifications. Elucidating this relationship is crucial for deciphering ADE mechanisms and developing safe interventions [10].

Previous reviews have mainly focused on the general virological and immunological mechanisms of ADE. In contrast, this review emphasizes the translational implications of antibody and Fc receptor biology in flavivirus infections, with particular attention to how antibody subclass, Fc glycosylation, FcγR polymorphisms, complement activation, and Fc engineering can inform safer vaccine development, antibody-based therapeutics, and individualized ADE risk assessment.

## 2. The Relationship Between Antibody Properties and ADE

### 2.1. The Influence of Antibody Types and Subtypes on ADE

The diverse roles mediated by distinct antibody populations in flavivirus ADE are summarized in Table 1. During DENV infection, non-neutralizing or sub-neutralizing IgG can mediate ADE of infection in cells expressing FcγRs and may contribute to ADE of disease by inducing pro-inflammatory cytokine production in primary human macrophages [11]. Furthermore, elevated IgG levels are commonly detected during secondary DENV infection and may be associated with an increased risk of antibody-mediated infection or disease enhancement, depending on antibody specificity, concentration, subclass, and Fc characteristics [11,12]. The induction of FcγR-mediated ADE of infection depends on the engagement of virus–antibody complexes with FcγRs. This receptor dependency is classically observed in vitro using K562 and U937 cell lines [13]. Clinical studies have also reported increased FcγRIIIa (CD16a) expression during acute dengue infection, which may facilitate FcγR-mediated viral uptake and contribute to both ADE of infection and downstream immunopathology [11,14].

Among the four human IgG subclasses, IgG1 and IgG3 have been most frequently implicated in ADE because of their relatively strong engagement of activating FcγRs [13,29]. The critical role of human IgG1 in ADE pathogenesis is primarily attributed to its high-affinity binding to FcγRIIIa [18]. Furthermore, IgG1 and IgG3 can effectively bind to FcγRI and FcγRIIa. In contrast, human IgG2 and IgG4 exhibit lower binding affinities for these receptors [15]. These FcγR-binding properties are principally determined by the IgG subclass, Fc glycosylation, and FcγR polymorphisms rather than by whether the Fab-mediated activity of an antibody is neutralizing or non-neutralizing. However, antibody concentration, epitope occupancy, and the size and geometry of virus–antibody immune complexes can influence multivalent FcγR engagement and receptor cross-linking. Thus, even a neutralizing antibody may promote ADE at sub-neutralizing concentrations if the resulting immune complexes permit productive FcγR-mediated viral uptake. Subsequent FcγR signaling and inflammatory responses may further contribute to ADE of disease. In murine experimental models, IgG2a or its allelic variant IgG2c—expressed by different mouse strains, such as BALB/c and C57BL/6, respectively—shares certain functional properties with human IgG1, including relatively strong engagement of activating FcγRs. In the experimental system reported by Byrne et al., mouse IgG2a showed strong binding to FcγRI on U937 cells [12], although human and murine IgG subclasses and FcγR systems are not directly equivalent. Interestingly, a recent study suggested that certain ADE-associated IgGs might also exploit alternative cellular entry pathways, such as B-cell receptor (BCR)-mediated ADE of infection [30].

Beyond homologous DENV infections, cross-reactive IgG antibodies elicited by prior heterologous flavivirus exposure may mediate ADE of infection in experimental systems. In mouse models, pre-existing DENV- or WNV-reactive antibodies have been shown to increase ZIKV infection, viral burden, and, under some experimental conditions, disease severity or mortality [31,32]. However, convincing epidemiological evidence that cross-flavivirus-reactive antibodies increase the risk of ADE of disease in humans remains limited. Santos-Peral et al. showed that prior vaccination against tick-borne encephalitis virus (TBEV) skewed the antibody response to subsequent yellow fever vaccination toward cross-reactive IgG targeting the highly conserved fusion loop epitope (FLE) within domain II (DII) of the flavivirus E protein. These antibodies showed the capacity to enhance DENV infection in an experimental assay [9]. Similarly, antibodies induced by a Japanese encephalitis virus (JEV) vaccine enhanced infection in vitro, but the same vaccine remained protective in pigs. This apparent discrepancy further illustrates that experimental ADE of infection does not necessarily translate into ADE of disease in vivo, and the clinical relevance of JEV-associated enhancement in humans remains uncertain [8,33]. Thus, the available evidence supports cross-flavivirus ADE of infection as a consideration in vaccine safety assessment, but does not establish a population-level risk of ADE of disease in humans. To reduce the potential for FLE-mediated ADE of infection, current ZIKV and WNV vaccine designs increasingly prioritize less cross-reactive antigenic targets, including E protein domain III (EDIII) and non-structural protein 1 (NS1) [33,34,35,36].

In clinical practice, the IgM/IgG ratio constitutes an established diagnostic benchmark for differentiating primary from secondary DENV infections [11,21]. In contrast to IgG, there is currently no direct evidence implicating IgM antibodies in the induction of flavivirus ADE. Nevertheless, studies highlight that early-emerging, virus-specific IgM confers essential protection during primary Zika virus infection. These antibodies not only directly neutralize the virus but can also suppress IgG-mediated ADE of infection and may thereby reduce the risk of ADE of disease [21]. Mechanistically, the high-avidity pentameric structure of IgM may competitively occupy viral epitopes and sterically limit the formation or FcγR-mediated uptake of enhancing IgG–virus complexes. Moreover, because IgM does not engage the FcγRs that classically mediate ADE of infection, it may instead promote viral neutralization and clearance through C1q recruitment and classical complement activation [20,37,38].

Beyond the classical IgM and IgG responses, emerging evidence implicates IgA and IgE isotypes in the modulation of DENV ADE [21,23]. Wegman et al. engineered and compared IgG and IgA isotypes of DENV-specific monoclonal antibodies (mAbs). They demonstrated that isotype switching preserved antigen-binding and neutralization capacities, but the IgA variant effectively antagonized the ability of its IgG counterpart to mediate ADE of infection [21]. Building on this, an mRNA-delivered IgA1 mAb has been shown to robustly neutralize DENV without triggering detectable ADE of infection, highlighting a promising and safe therapeutic avenue [39].

In contrast, the role of IgE is largely linked to hypersensitivity. IgE may indirectly exacerbate ADE of disease through mast cell and basophil degranulation, leading to the release of vasoactive mediators and increased vascular permeability [23]. However, whether IgE directly contributes to ADE of infection by facilitating DENV replication, or instead acts as an indirect marker or modulator of disease severity, remains unclear. Although serum IgE levels have been associated with enhanced viral replication, current evidence is largely correlative and does not establish an IgE-specific causal mechanism [40].

Overall, although different antibody isotypes have been implicated in DENV ADE, their respective contributions to ADE of infection and ADE of disease require further clarification. Additionally, the involvement of non-IgG antibodies (such as IgM, IgA and IgE) in ZIKV and WNV ADE remains poorly understood and requires further investigation.

### 2.2. The Effect of Fc Region Modification on ADE

Beyond antibody isotypes and subclasses, the amino acid sequence and glycosylation of the Fc region influence antibody effector function and FcγR engagement (Table 2). Fc fucosylation refers to the presence of a core fucose residue on the N297-linked Fc glycan, whereas afucosylation refers to its absence. Loss of this core fucose increases the affinity of IgG for FcγRIIIa and can enhance FcγRIIIa-dependent effector functions, including antibody-dependent cellular cytotoxicity. In DENV infection, however, increased FcγRIIIa engagement by afucosylated IgG has also been associated with ADE of infection and greater disease severity [41,42,43]. Other targeted Fc modifications can reduce Fc receptor binding and thereby limit ADE of infection and its potential contribution to ADE of disease [44].

In DENV infection, afucosylated IgG shows increased FcγRIIIa binding and has been associated with enhanced susceptibility to ADE of infection and greater disease severity [41,42]. By contrast, core fucosylation reduces FcγRIIIa affinity and may limit these effects [43]. To counter ADE, Yang et al. demonstrated that adding plant-specific xylose and α1,3-fucose into the N-glycosylation completely blocks detectable DENV ADE of infection and attenuated ZIKV ADE of infection, while preserving neutralizing and ADCC functions [45,46].

**Figure 1:**
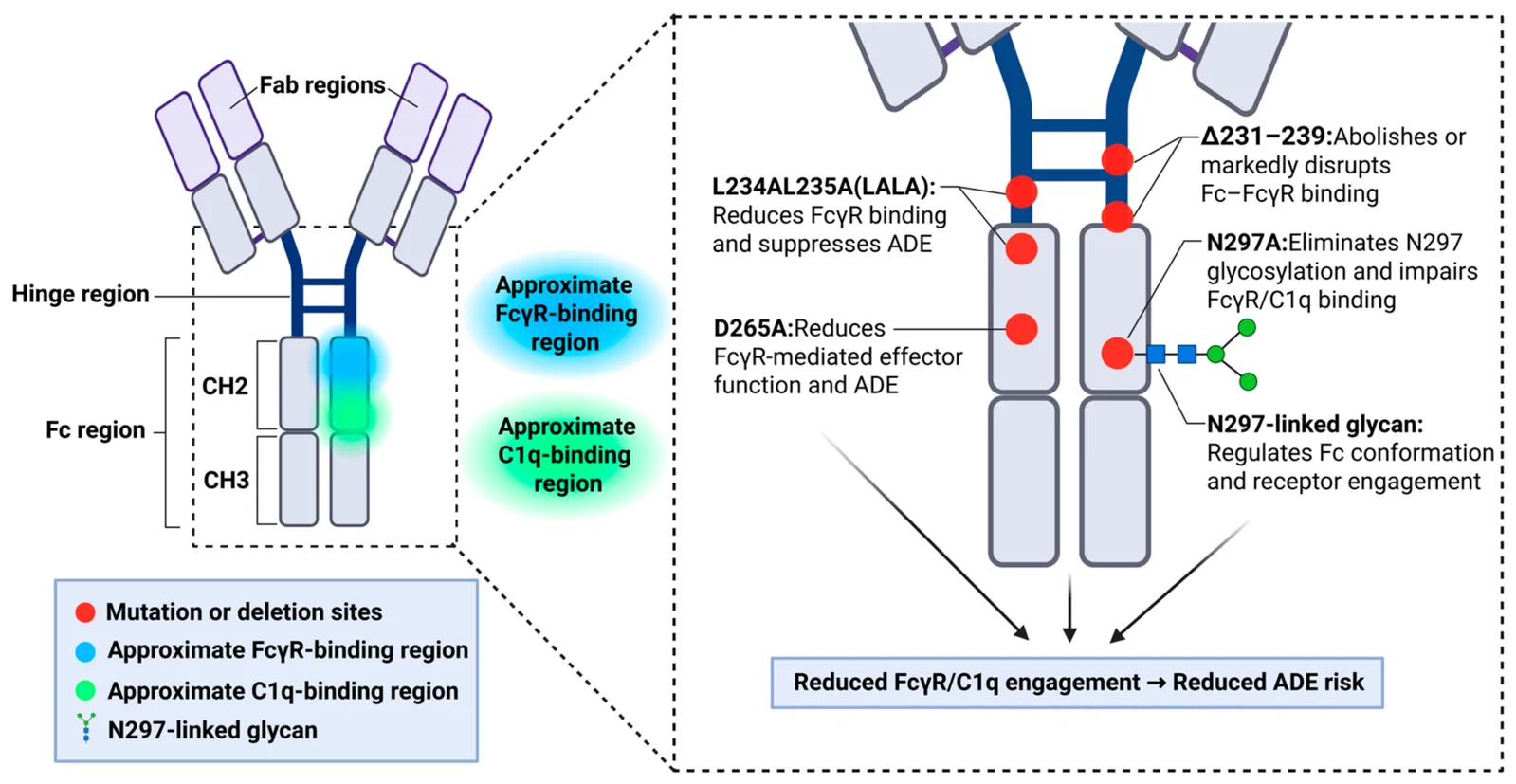
Fc-region engineering sites associated with reduced ADE of infection. The schematic shows the approximate locations of deletion 231–239, L234A/L235A (LALA), D265A, N297A, the N-linked glycan at N297, and the FcγR- and C1q-interacting regions. These modifications reduce FcγR and/or C1q engagement in the cited experimental systems. Residue positions and interaction interfaces are schematic, and numbering follows the cited studies. Created in BioRender by Yiling Li (2026) https://BioRender.com/mc4m9hu.

Additionally, targeted amino acid substitutions or deletions in the Fc region can reduce ADE of infection by disrupting Fc receptor interactions (Figure 1). For instance, the N297A substitution eliminates Fc N-glycosylation and consequently impairs binding to FcγRs and C1q [46]. The LALA substitutions (L234A/L235A) similarly reduce FcγR binding, including binding to FcγRIIa [46]. Lu et al. further demonstrated that both the LALA substitutions and deletion of residues 231–239, a region involved in Fc–FcγR interactions, eliminated detectable ADE of infection in their experimental system [44]. Consistent findings have been reported for the LALA, N297A, and D265A variants [42]. Together, these Fc-engineering strategies provide a mechanistically grounded approach to retaining antibody neutralization while reducing Fc receptor-mediated viral uptake and the potential downstream risk of ADE of disease.

Building on these modification strategies, Fc-region engineering is similarly effective in suppressing FcγR-mediated ADE of infection by ZIKV. ZIKV ADE of infection may occur in individuals with pre-existing, non-neutralizing antibodies from a prior flavivirus infection. However, unlike DENV, the contribution of natural Fc glycosylation to ZIKV ADE of infection appears limited. Bournazos et al. (2021) reported that patients with ZIKV infection did not exhibit elevated levels of afucosylated anti-ZIKV antibodies (anti-E and anti-NS1), regardless of prior DENV exposure [50]. Instead, amino acid sequence modifications remain the principal approach to prevent ADE. Consistent with the DENV studies, Nkolola et al. (2024) developed a therapeutic monoclonal antibody (ZIKV-117-LALA-YTE) incorporating the LALA mutation [48]. This modification eliminates Fcγ receptor binding, thereby significantly reducing the risk of FcγR-mediated ADE of infection and, potentially, ADE of disease.

## 3. Fc Receptor Subtypes and Their Roles in ADE of Infection and Disease

### 3.1. Classification and Expression of FcR

As outlined in Table 1, the interaction of virus–antibody complexes with Fc receptors is central to ADE of infection, while the resulting Fc receptor signaling may additionally contribute to ADE of disease [51]. FcγRs are primarily classified into activating and inhibitory receptors. Activating receptors contain immunoreceptor tyrosine-based activation motifs (ITAMs) and include FcγRI (CD64, found on monocytes, macrophages, neutrophils and mast cells), FcγRIIa (CD32a, found on monocytes, macrophages, neutrophils, dendritic cells, basophils, mast cells, eosinophils, and platelets), FcγRIIc (CD32c, found on NK cells), and FcγRIIIa (CD16a, found on monocytes, macrophages, and NK cells) [16]. Conversely, the inhibitory receptors such as FcγRIIb (CD32b, found on monocytes, macrophages, B cells, dendritic cells, basophils and neutrophils) contain an immunoreceptor tyrosine-based inhibition motif (ITIM) [16]. Additionally, FcγRIIIb (CD16b) is a glycolipid-phosphatidylinositol (GPI)-anchored receptor on neutrophils and basophils; lacking intrinsic signal-transduction capacity, it relies primarily on other immune receptors or helper chains for signaling [17].

At steady state, spontaneous receptor cross-linking is prevented because only high-affinity FcγRI can bind monomeric IgG [15]. During infection, however, multivalent virus–antibody immune complexes cross-link both high- and low-affinity activating receptors (FcγRI, FcγRIIa and FcγRIIIa), thereby initiating FcγR-mediated ADE of infection and downstream signaling that may contribute to ADE of disease [52]. While activating FcγRs promote ADE of infection and pro-pathogenic signaling, the inhibitory receptor FcγRIIb may counterbalance these effects [42,52,53,54,55]. Consequently, cellular susceptibility to ADE depends on the integrated signaling and expression ratio of activating versus inhibitory FcγRs. For instance, mononuclear phagocytes heavily express FcγRIIa and serve as primary targets for ADE of infection, whereas the predominant expression of FcγRIIb on B cells confers relative resistance [30]. Ultimately, this activating-to-inhibitory balance determines the clinical trajectory, dictating whether an infection—such as dengue—resolves as a self-limiting fever or progresses to severe disease.

As previously mentioned, IgM antibodies may inhibit ADE of infection. This protective effect is likely driven by two main factors: receptor specificity and structural constraints. First, the Fc region of IgM preferentially binds to FcμR (expressed on B cells, T cells and NK cells) and complement component C1q, rather than to the FcγRs that classically mediate ADE of infection. Therefore, the FcγR-mediated viral endocytosis will not be initiated [20]. In addition, the intracellular domain of FcμR does not contain typical ITAMs or ITIMs. Signal transduction mainly relies on the His residues in its transmembrane region and the Tyr and/or Ser residues in its cytoplasmic tail. After binding to IgM, it can mediate immune regulation and B cell activation, further promoting immune clearance and regulating B cell responses [38]. Second, the massive pentameric structure of IgM creates steric hindrance, effectively preventing it from engaging cell-surface FcγRs in the manner that monomeric IgG does, thereby precluding viral phagocytosis [20,37,38].

Similarly, IgA antibodies may inhibit ADE of infection, which is primarily attributed to their low-affinity binding to FcαR (CD89; expressed on monocytes, macrophages, neutrophils, eosinophils and dendritic cells), an interaction insufficient to drive efficient viral entry [11,22]. On the other hand, although IgA immune complexes (such as IgA bound to pathogens) can trigger the classical ITAM signaling pathway when they interact with FcαR, activating immune cells and initiating pro-inflammatory responses to eliminate pathogens [56]. Wegman et al. clearly showed that when DENV-IgA complexes interact with cells, they do not induce the production of pro-inflammatory cytokines, and may even slightly inhibit inflammation [11]. Therefore, it is more likely to attribute the protective effect of IgA to its physical and chemical properties (affinity, structure) and competitive antagonism of IgG-mediated ADE, rather than the complex intracellular signal negative regulation.

Unlike the reported protective roles of IgM and IgA against ADE of infection, the contribution of IgE remains less clearly defined and may primarily involve mechanisms associated with ADE of disease. This pathogenic process is predominantly driven by the engagement of IgE with its high-affinity receptor, FcεRI. FcεRI is broadly expressed across various innate immune cells (including monocytes, macrophages, dendritic cells, eosinophils, and platelets). However, it is currently believed that the interaction between IgE and FcεRI mainly contributes to the disease ADE by cross-linking and activating mast cells and basophils, releasing vasoactive mediators such as histamine and leukotrienes, which further exacerbate vascular permeability and inflammatory responses, thereby possibly increasing the clinical severity of yellow fever infections and other viral infections. Conversely, the exact contribution of the low-affinity receptor, FcεRII, which is distributed on B cells, monocytes, macrophages, dendritic cells, eosinophils, and platelets, to the ADE phenomenon remains elusive and warrants further investigation [23,24,25].

FcRn does not directly mediate ADE of infection. Instead, it transports maternal IgG across the placenta and prolongs IgG persistence in the circulation [26]. Over time, these maternally derived antibodies naturally decay to sub-neutralizing levels. Because neonatal monocytes express activating receptors (FcγRI and FcγRIIa) at levels comparable to those in adults, these residual, non-neutralizing antibodies can readily form immune complexes during a primary DENV infection, thereby facilitating ADE of infection and potentially increasing the risk of ADE of disease in infants [28].

### 3.2. FcγR Polymorphisms and Susceptibility to ADE

FcγR polymorphisms may modify ADE susceptibility by altering IgG-subclass binding and the balance between activating and inhibitory signaling. FcγRIIa H131R is the most studied variant in dengue. A Pakistani case–control study associated the H allele with clinical dengue and DHF/DSS, but the cohort was small, included participants aged 1–24 years, and did not determine the infecting serotype [57]. Studies in Cuba and Vietnam reported estimates in the same direction, although statistical support was inconsistent [58,59]. In contrast, a Mexican study associated the H allele with protection from complicated dengue [60]. Differences in sample size, age, serotype, infection history, ancestry, and disease definitions may contribute to these divergent findings [57,58,59,60]. The functional difference between the H131 and R131 variants is best established for IgG2 binding, and the conflicting epidemiological results should not be attributed solely to presumed differences in the predominant IgG subclass among populations [15,28]. Although population-level variation in IgG subclass profiles may contribute to the context-dependent associations of FcγRIIa H131R with dengue outcomes, this interpretation remains hypothetical. Direct functional studies that jointly assess FcγRIIa genotype, IgG subclass distribution, immune-complex handling, and ADE-related outcomes are still lacking, and this proposed relationship requires experimental validation. Other candidate variants include FcγRIIIa V158F, which alters receptor affinity for IgG1 and IgG3, and FcγRIIb I232T, which impairs inhibitory receptor function [15,61]. However, direct epidemiological evidence linking either variant to ADE of infection or ADE of disease in humans remains limited.

### 3.3. The FcγR Signaling Pathway and Viral Replication

**Figure 2:**
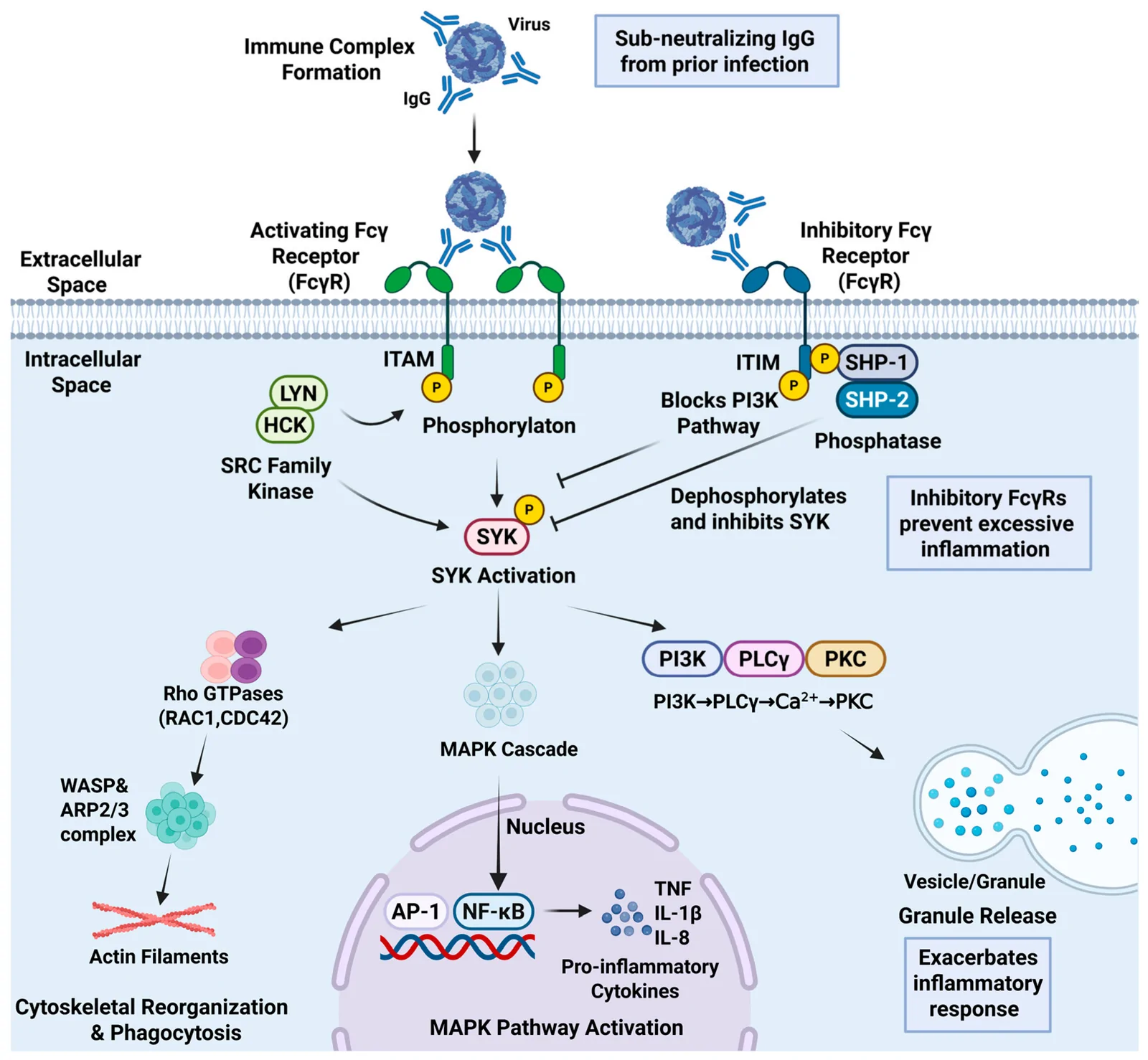
FcγR-mediated signaling during flavivirus antibody-dependent enhancement. Following secondary infection or exposure to cross-reactive antibodies, non-neutralizing or sub-neutralizing IgG antibodies form immune complexes with flaviviruses. These complexes bind to activating Fcγ receptors (FcγRI, FcγRIIa, FcγRIIIa) on target cells, such as monocytes and macrophages, leading to receptor cross-linking. This process triggers phosphorylation of ITAMs by SRC family kinases, such as LYN and LCK, followed by the recruitment and activation of SYK kinase. Activated SYK subsequently initiates downstream signaling cascades, including the PI3K-PLCγ-Ca^2+^-PKC and MAPK pathways. These events promote cytoskeletal reorganization, phagocytosis, and the release of pro-inflammatory cytokines, such as TNF, IL-1β and IL-8, as well as chemokines. In contrast, the inhibitory receptor FcγRIIb recruits SHP1/SHP2 phosphatases via its ITIM, thereby dampening activation signals. Arrows with pointed heads indicate activation; bars indicate inhibition. Different colors are used to distinguish different proteins for clarity. Created in BioRender by Yiling Li (2026) https://BioRender.com/0s2knau.

The fundamental prerequisite for FcγR-mediated ADE of infection is the formation of immune complexes between viruses and non-neutralizing or sub-neutralizing IgG antibodies, which are typically generated during a primary infection (Figure 2). Upon secondary exposure, these immune complexes simultaneously engage multiple FcγRs on target cells. This multivalent binding triggers receptor aggregation and cross-linking, driving the recruitment of SRC family kinases (such as LYN, LCK, HCK, and FGR). These kinases subsequently phosphorylate the ITAMs located on the intracellular domains of the activated FcγRs [62,63,64]. The phosphorylated ITAMs then serve as docking sites for spleen tyrosine kinase (SYK), leading to its recruitment, autophosphorylation, and activation, which in turn orchestrates multiple downstream signaling cascades [65].

Specifically, activated SYK diverges into three primary effector pathways. First, it activates the PI3K-PLCγ-Ca^2+^-PKC axis, which stimulates degranulation in granulocytes to clear pathogens; this process inadvertently exacerbates the inflammatory response [66,67]. Second, SYK drives the cytoskeletal rearrangements essential for phagocytosis. This is mediated via the activation of Rho GTPases (e.g., RAC1, RAC2, CDC42), which stimulate the ARP2/3 complex and WASP proteins to promote actin polymerization, ultimately facilitating the internalization of virus–antibody complexes [68]. Third, SYK activation triggers the mitogen-activated protein kinase (MAPK) signaling cascade [69]. Activated MAPKs then translocate to the nucleus to activate transcription factors such as AP-1 and NF-κB, culminating in the robust expression and secretion of chemokines and pro-inflammatory cytokines (e.g., TNF, IL-1β, IL-8) [70,71].

To counterbalance these potent activation signals, the immune system utilizes inhibitory FcγRs to maintain physiological homeostasis. Upon receptor cross-linking, the ITIMs of inhibitory FcγRs are phosphorylated by SRC kinases, thereby recruiting the phosphatases SHP1 and SHP2 [72]. SHP1 negatively regulates the cascade by dampening the PI3K-PLCγ pathway, whilst SHP2 directly dephosphorylates and deactivates key kinases including SYK [73]. Together, these mechanisms act as a critical brake to prevent excessive inflammation.

**Figure 3:**
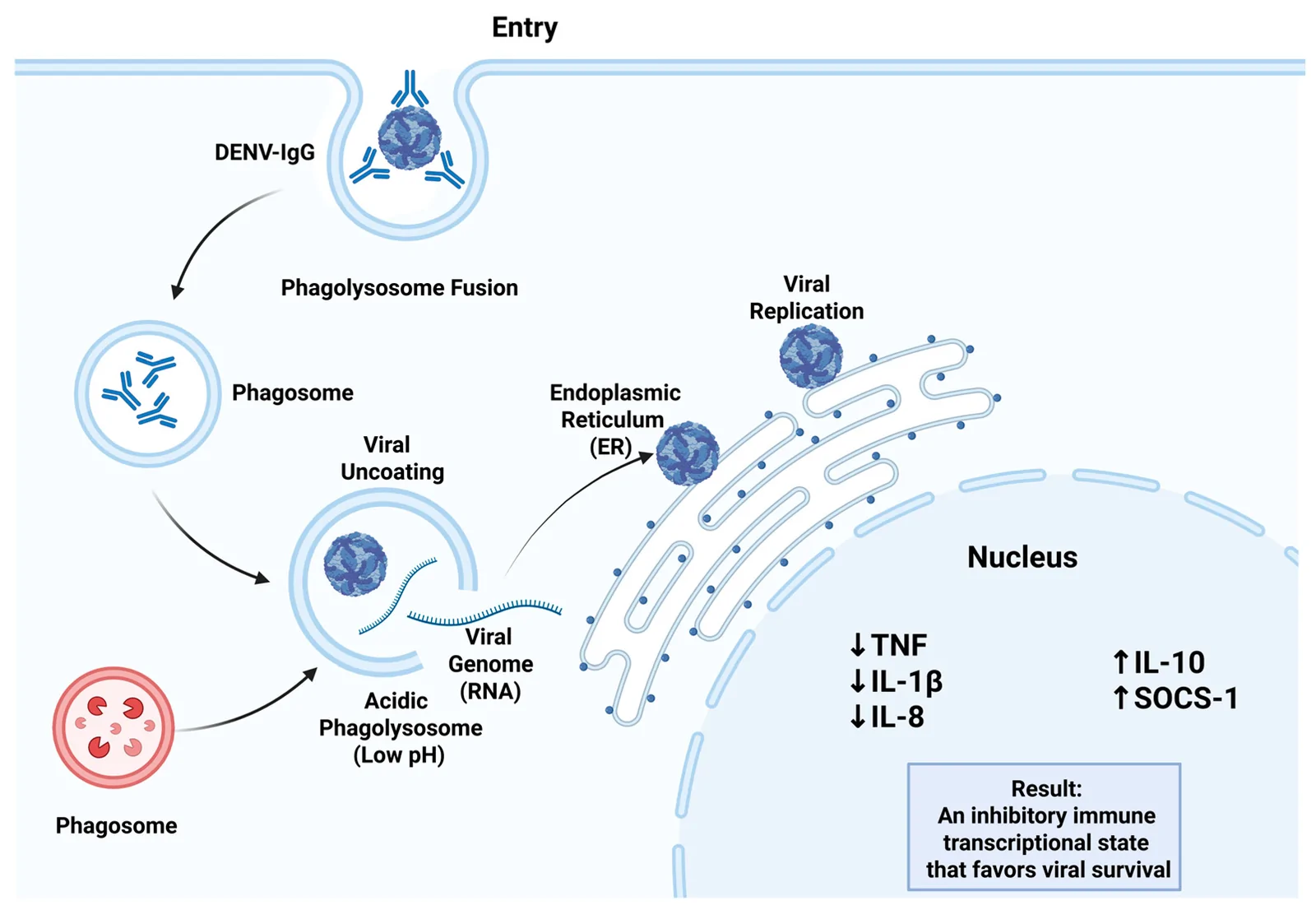
Intracellular events following FcγR-mediated uptake of DENV–antibody complexes. Following FcγR-mediated phagocytosis of DENV–antibody complexes, the virus is internalized into acidified phagosomes. The low-pH environment induces conformational changes in the viral E protein, facilitating fusion with the lysosomal membrane and subsequent release of the viral genome into the cytoplasm for replication. Notably, macrophages undergoing antibody-enhanced DENV infection exhibit a distinct transcriptional profile characterized by suppressed inflammatory cytokine expression, downregulated interferon signaling, and mitochondrial dysfunction. In contrast, uninfected bystander macrophages display an activated phenotype, with upregulated antigen-presenting and interferon-stimulated genes. Upward arrows indicate increased expression; downward arrows indicate decreased expression. Created in BioRender by Yiling Li (2026) https://BioRender.com/t4e4l71.

While the mechanism of FcγR-mediated phagocytosis is well established, the subsequent intracellular events remain complex. Following endocytosis, DENV–antibody complexes enter acidic phagosomes, where the low pH triggers a conformational change in the viral E protein. This facilitates fusion with the lysosomal membrane, enabling viral genome release and replication (Figure 3) [52]. Concurrently, this internalization promotes an immunosuppressive state, characterized by the downregulation of Th1-type cytokines (IL-12, IFN-γ, TNF-α) and the upregulation of IL-10 and cytokine signaling suppressor of type 1 (SOCS-1) [74,75,76].

Paradoxically, this immunosuppressive transcriptional profile seemingly contradicts the MAPK-driven pro-inflammatory response described earlier. However, Hardy et al. (2025) recently resolved this discrepancy. They demonstrated that under ADE-of-infection conditions, DENV-infected macrophages exhibit an intrinsically suppressed inflammatory profile, downregulated interferon signaling, and mitochondrial dysfunction, which is independent of viral RNA load. In stark contrast, uninfected “bystander” macrophages display a highly activated phenotype with upregulated antigen-presenting and interferon-stimulated genes [77]. Thus, the broader inflammatory storm is primarily driven by uninfected bystander cells, whereas cells undergoing antibody-enhanced infection suppress their intrinsic to evade clearance.

**Figure 4:**
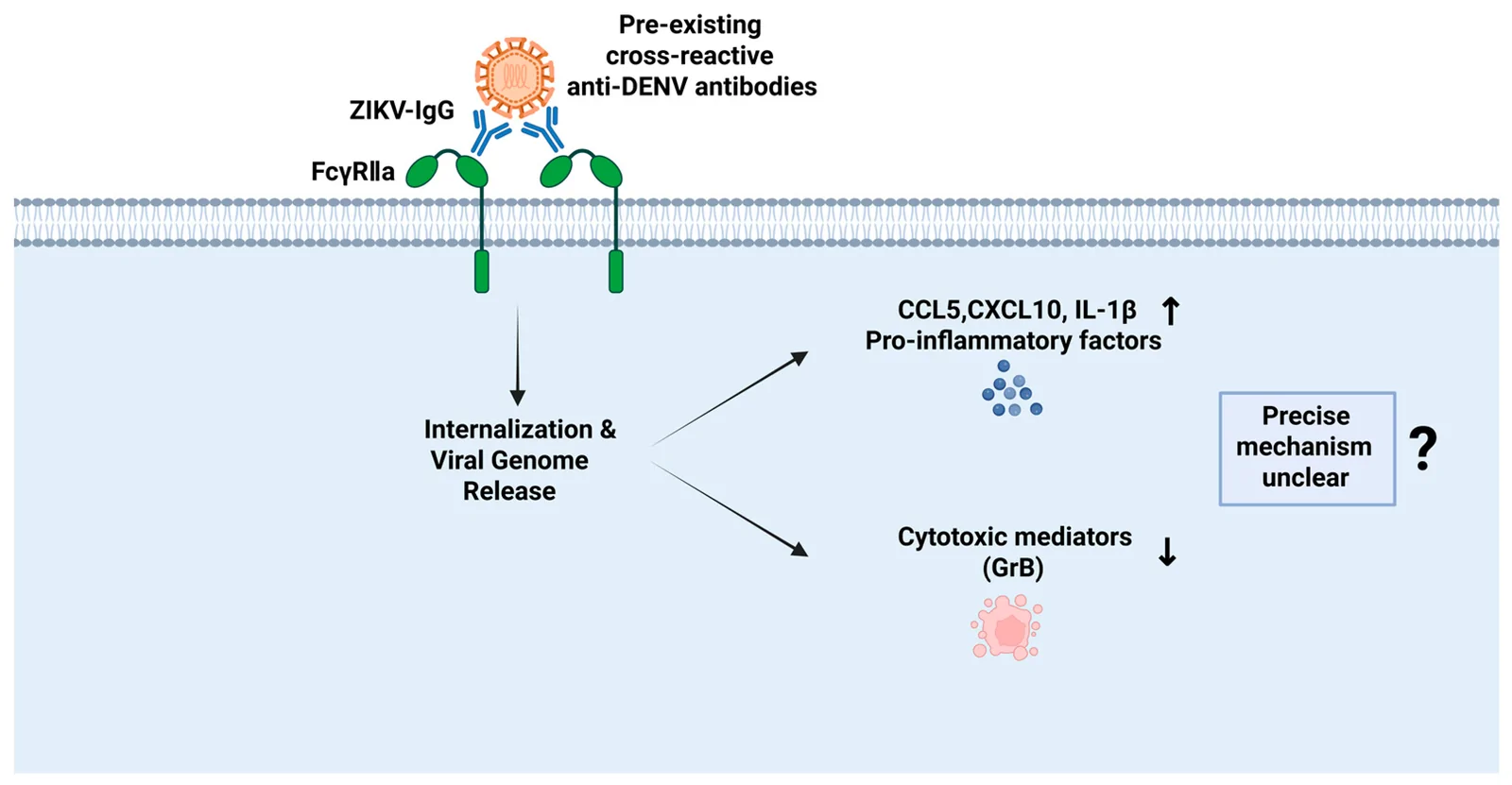
Experimental heterologous ADE of ZIKV infection mediated by DENV-induced cross-reactive antibodies. Cross-reactive IgG antibodies induced by prior DENV infection recognize ZIKV and form immune complexes that bind to FcγRIIa on target cells. This interaction facilitates ADE of infection through viral internalization and genome release, accompanied by the secretion of pro-inflammatory factors such as CCL5, CXCL10, and IL-1β, together with reduced expression of cytotoxic mediators such as granzyme B (GrB). However, the precise molecular pathways underlying these immune alterations remain incompletely defined. Upward arrow indicates increased expression; downward arrow indicates decreased expression. Created in BioRender by Yiling Li (2026) https://BioRender.com/tf6r2t5.

Experimental heterologous ADE of ZIKV infection has been demonstrated in the presence of pre-existing DENV- or WNV-reactive antibodies [29,30]. In a DENV antibody-enhanced ZIKV mast-cell model, increased viral replication was accompanied by elevated secretion of pro-inflammatory mediators, including CCL5, CXCL10, and IL-1β, and reduced expression of granzyme B [78] (Figure 4). ADE of WNV infection has also been demonstrated using sub-neutralizing concentrations of WNV-specific anti-E monoclonal antibodies in FcγR-expressing cells and in vivo, although the reported increase in infection was not accompanied by increased lethality or an altered disease phenotype [79]. Thus, experimental evidence supports ADE of infection for both ZIKV and WNV, but the antibody source and experimental context differ, and evidence that heterologous DENV-reactive antibodies enhance WNV infection remains limited.

### 3.4. Interaction Between the Fc Region of Antibodies and the Complement System

The complement system, a crucial network of serum and membrane-bound proteins, plays a pivotal role in antiviral immunity. Upon infection, it is classically activated via three distinct routes: the classical (triggered by antigen–antibody complexes engaging C1q), alternative, and mannose-binding lectin (MBL) pathways. All three cascades converge on the cleavage of C3, culminating in the assembly of the membrane attack complex (MAC; C5b-9), which directly lyses infected cells or enveloped viruses [80]. Furthermore, intermediate cleavage fragments generated during these cascades exhibit potent anti-flavivirus activity. For instance, C1q and MBL can directly bind to the E protein of DENV and WNV to neutralize viral infectivity [81,82], while opsonins such as C3b and C4b engage complement receptors to facilitate viral phagocytosis and clearance [83].

The complement system can markedly inhibit flavivirus ADE of infection [84]. Yamanaka et al. demonstrated that monoclonal antibodies enhanced DENV infection in heat-inactivated serum; the addition of intact complement completely abrogated this enhancement in a C1q- and C3-dependent manner [85]. Similarly, Mehlhop et al. established that high-affinity binding of C1q to anti-WNV antibodies was sufficient to suppress WNV ADE of infection [79]. Two related mechanisms may account for these observations: steric competition for Fc binding and alteration of the stoichiometric requirements for neutralization. First, the C1q- and FcγR-interacting regions on the IgG Fc domain partially overlap. C1q binding to virus–antibody complexes may therefore restrict FcγR access to the Fc region and reduce FcγR-mediated viral uptake [79,86]. Second, C1q can lower the antibody occupancy required for viral neutralization. For IgG subclasses that bind C1q efficiently, this shift may reduce the neutralization threshold below the antibody occupancy associated with enhancement [86]. Thus, an antibody concentration that remains sub-neutralizing and permits ADE of infection in the absence of C1q may become sufficient for neutralization when C1q is present. From this perspective, C1q-mediated enhancement of neutralization and inhibition of ADE of infection are closely related consequences of the same change in antibody stoichiometry rather than entirely separate mechanisms [86].

These findings suggest that complement availability and C1q-binding capacity should be considered when evaluating the balance between antibody-mediated neutralization and enhancement [79,84,86]. Experimental studies provide proof of principle that restoring complement activity can suppress ADE of infection under complement-deficient conditions. Mehlhop et al. found that the absence of functional C1q increased enhancement, whereas the addition of C1q restored its inhibitory effect [79]. Yamanaka et al. confirmed that the level of complement could determine whether the antibody manifested as neutralization or enhancement [85]. These observations raise the possibility that preserving or selectively augmenting C1q-dependent antiviral activity could inform future complement-directed interventions. However, C1q supplementation has not been established as a clinical strategy, and systemic manipulation of complement could alter immune-complex clearance, inflammation, and tissue injury. Complement-informed intervention should therefore be regarded as a research direction that requires validation in physiologically relevant animal models and clinical studies, rather than as an immediately applicable approach for preventing ADE.

In contrast to its protective role against flaviviruses, complement paradoxically exacerbates ADE in other viral infections, such as HIV, Ebola and SARS-CoV-2. In these contexts, C1q binds the virus–antibody complex, which subsequently engages host complement receptors. This alternative entry route not only increases intracellular viral load, thereby mimicking canonical ADE, but also triggers robust inflammatory cytokine release [9,87].

In summary, complement acts as a double-edged sword in viral ADE. While it suppresses flavivirus ADE of infection through multifaceted mechanisms, it promotes infection in other viral families. Unraveling the distinct molecular mechanisms governing this virus-specific dichotomy is imperative, as targeted modulation of complement pathways holds significant therapeutic promise for circumventing ADE.

## 4. Prophylactic Flavivirus Vaccines and ADE Risk

### 4.1. Current Status of Flavivirus Vaccines

Vaccination is the principal strategy for preventing flavivirus infection and disease [87]. Three tetravalent dengue vaccines, CYD-TDV (Dengvaxia), TAK-003 (Qdenga), and Butantan-DV, have received licensure in one or more jurisdictions [7]. CYD-TDV uses a yellow fever virus 17D backbone expressing the prM and E proteins of the four DENV serotypes. TAK-003 is based on an attenuated DENV2 backbone and expresses the prM and E proteins of the other three serotypes. Butantan-DV contains attenuated components representing all four serotypes. These vaccines differ in antigen composition, replication kinetics, and the balance of immunity induced against individual serotypes.

The experience with CYD-TDV shows that baseline immunity is an important determinant of dengue vaccine safety. Among recipients without previous DENV infection, vaccination was associated with an increased subsequent risk of hospitalization and severe dengue. Its use was therefore restricted to individuals with evidence of previous infection [88]. This observation is consistent with vaccination acting immunologically as a primary exposure in seronegative recipients, although the mechanisms linking vaccine-induced antibodies to later disease remain incompletely defined. Vaccine evaluation should therefore consider baseline serostatus, serotype-specific immunity, antibody durability, and clinical outcomes after natural exposure.

No vaccine is currently licensed for human use against ZIKV or WNV. Candidates based on inactivated virus, viral vectors, protein subunits, and nucleic acids have entered early clinical development [89,90]. Previous flavivirus exposure may alter responses to these vaccines through cross-reactive immunity. Experimental ADE of infection should therefore be assessed, but it should not be interpreted as evidence of ADE of disease in humans without corresponding clinical or epidemiological data.

### 4.2. ADE Risk Across Vaccine Platforms

ADE risk reflects antigen composition, epitope presentation, baseline immunity, and antibody durability rather than vaccine platform alone. Live-attenuated and chimeric vaccines induce broad humoral and cellular responses, but their individual components may differ in replication and immunogenicity. In tetravalent dengue vaccines, an imbalanced response may provide stronger protection against some serotypes than others. Waning antibody concentrations may also create a sub-neutralizing range during subsequent infection, particularly in recipients without previous DENV immunity.

Protein subunit vaccines allow for more selective antigen presentation. Constructs based on EDIII or NS1 can reduce exposure to the cross-reactive epitopes found in prM and the E protein fusion loop [91,92,93,94]. A ZIKV EDIII-Fc vaccine increased antigen immunogenicity and reduced experimental ADE of infection [95]. However, Fc-containing vaccine constructs require evaluation of FcγR-dependent uptake and effector function. NS1-based vaccines do not induce antibodies against virion-surface proteins and are therefore less likely to mediate classical ADE of infection. Potential cross-reactivity of some NS1 antibodies with platelets or endothelial cells remains a separate safety concern.

VLPs preserve conformational epitopes without supporting viral replication. Their antibody profile depends on particle composition and maturation. Partially mature particles may expose prM and fusion-loop epitopes, whereas engineered VLPs can exclude these regions. A quadrivalent DENV VLP lacking prM induced balanced neutralizing responses and reduced prM-associated ADE of infection in experimental models [96]. WNV VLPs displaying EDIII similarly excluded the fusion loop and retained protective immunogenicity [97].

DNA and RNA platforms permit direct modification of antigen sequence and expression. A DENV DNA vaccine based mainly on NS-derived T-cell epitopes was designed to reduce reliance on virion-binding antibodies [98]. A circular RNA vaccine encoding ZIKV EDIII-Fc and NS1 induced neutralizing responses without detectable DENV cross-ADE of infection in mice [93]. Stabilization of the ZIKV E protein through G5C and G102C substitutions masked the fusion loop and reduced experimental cross-enhancement [91]. D87N and L107F substitutions in WNV and ZIKV prM/E vaccines also reduced heterologous ADE of infection while preserving homologous neutralization [99].

These studies support several antigen-design principles: removal of prM, masking of the fusion loop, stabilization of E protein dimers, and enrichment of EDIII or selected non-structural antigens. Their effects should be tested across a range of antibody concentrations and against both homologous and heterologous viruses. Cellular enhancement assays can identify ADE of infection, whereas claims regarding ADE of disease require animal pathology, clinical, or epidemiological evidence.

### 4.3. Systems Serology-Based Risk Stratification

Neutralizing antibody titers do not capture the full range of Fc-dependent functions that may influence protection or enhancement. Systems serology integrates antigen specificity, antibody isotype and subclass, Fc glycosylation, Fc receptor binding, and effector function [100]. Multiplexed Fc arrays can measure antigen-specific subclasses and FcγR-binding profiles across large antigen panels using small sample volumes [101]. Flavivirus panels could include E, EDIII, prM, and NS1 antigens from homologous and heterologous viruses. Functional measurements may include antibody-dependent cellular phagocytosis (ADCP), antibody-dependent neutrophil phagocytosis (ADNP), antibody-dependent cellular cytotoxicity or NK-cell activation (ADCC/ADNKA), and antibody-dependent complement deposition (ADCD).

Dias et al. applied this approach to plasma collected before secondary DENV3 infection in a Nicaraguan pediatric cohort. Children who remained asymptomatic had stronger E- and NS1-specific Fc responses than those who developed symptomatic disease. ADCP, NK-cell activation, ADCD, and Fc polyfunctionality were associated with protection. E- and NS1-specific ADCD was also linked to complement-mediated lysis of virions and infected cells. A multivariable model combining E-specific IgG4 and Fc functions with NS1-specific IgG and ADCD distinguished inapparent from symptomatic infection [30].

Protective and enhancing responses are unlikely to be defined by a single Fc measurement. Durable neutralization combined with coordinated Fc-mediated clearance may indicate protection. Weak or waning neutralization, prM- or fusion loop-directed cross-reactivity, strong activating FcγR engagement, and increased infection at sub-neutralizing concentrations may indicate enhancement potential. Interpretation depends on antibody concentration, antigen specificity, Fc glycosylation, and immune-complex geometry. Fc profiling should therefore be assessed together with neutralization, ADE-of-infection assays, longitudinal measurements, and clinical outcomes.

Clinical application will require standardized antigen panels, reference sera, sampling schedules, controls, and acceptance criteria. Assays should be formally qualified using predefined performance criteria, including analytical precision, dynamic range, and intra- and inter-run variability, with common reference standards used to support comparisons across batches and study sites [102]. Baseline serostatus, previous infection or vaccination, infecting serotype, age, and disease severity should be recorded. Reporter systems may reduce the donor variability associated with primary-cell assays. Harmonized protocols for sample collection, processing, storage, and freeze–thaw cycles are also needed to preserve complement activity and minimize pre-analytical variation in antibody glycosylation measurements. Fc arrays could be used for initial cohort-wide screening, followed by targeted functional and virological testing. Predictive signatures require prospective validation in independent populations before they can inform participant stratification or vaccine decisions.

## 5. Therapeutic Interventions for Flavivirus Infection and ADE-Associated Disease

### 5.1. Viral Entry Inhibition and Antibody-Based Therapeutics

No specific antiviral drug is currently approved for DENV, ZIKV, or WNV. Therapeutic approaches can be classified according to whether they inhibit viral entry, block intracellular replication, or limit host-mediated pathology. The first two strategies primarily reduce viral burden, whereas host-directed treatment addresses the inflammatory and clinical manifestations of severe disease.

Entry inhibitors prevent viral attachment, membrane fusion, or FcγR-mediated uptake of virus–antibody complexes. Peptides derived from the E or M proteins can disrupt the conformational changes required for membrane fusion. DN59 inhibited infection by all four DENV serotypes in experimental systems [102,103].

Neutralizing monoclonal antibodies block viral entry through epitope-specific recognition. To reduce ADE of infection, candidate antibodies have been directed toward less cross-reactive epitopes or modified to reduce FcγR engagement [45,50,104]. EDIII contains serotype-specific neutralizing epitopes and is less conserved than the fusion loop [34,105,106,107]. NS1 antibodies do not neutralize virions directly but may promote infected-cell clearance or inhibit NS1-mediated pathology. Their potential reactivity with platelets and endothelial cells requires assessment [108].

Fc substitutions such as LALA, N297A, and D265A reduce FcγR binding and have inhibited ADE of infection in experimental models [46,48,50]. Fc glycosylation can also be modified to alter receptor affinity and effector activity [44,45,49]. These changes may reduce viral uptake but can also impair protective ADCC or phagocytosis. Fc-free nanobodies avoid classical FcγR-mediated ADE of infection, although Fc-fused nanobody constructs regain Fc receptor-dependent functions [109,110]. All antibody-based candidates should be tested across a concentration range for neutralization, Fc effector function, and enhancement.

### 5.2. Viral Replication Blockade

Direct-acting antivirals target viral processes after entry. The principal targets include the NS2B-NS3 protease, NS5 RNA-dependent RNA polymerase, and protein interactions required for replication-complex formation [111]. Their conservation across flaviviruses may permit activity against more than one virus.

JNJ-A07 disrupts the DENV NS3-NS4B interaction and suppresses replication across multiple serotypes [112]. Nucleoside analogues such as sofosbuvir inhibit DENV RNA synthesis in vitro, although incomplete efficacy and cellular toxicity have limited further development [113]. Other inhibitors of NS2B-NS3 or NS5 have shown activity against several flaviviruses in cell-based assays [114,115].

Cordycepin and berberine have also shown antiviral effects in experimental models. Berberine interferes with NS5-dependent replication and has activity against DENV, ZIKV, and chikungunya virus [116,117]. These compounds remain preclinical, and their therapeutic value depends on achievable exposure, selectivity, toxicity, and efficacy after symptom onset. Direct-acting agents do not depend on antiviral antibodies and are therefore not expected to induce classical FcγR-mediated ADE.

### 5.3. Host-Directed Immunomodulation and Supportive Care

Severe flavivirus disease may involve inflammation, vascular leakage, thrombocytopenia, shock, or organ dysfunction. Host-directed treatment aims to control these manifestations rather than ADE of infection itself [118].

Evidence for glucocorticoids in dengue remains inconclusive. Rupatadine has been evaluated as an antagonist of platelet-activating factor, and recombinant human IL-11, anti-D immunoglobulin, eltrombopag, doxycycline, and papaya-derived products have been studied for thrombocytopenia. The supporting evidence is heterogeneous, and these interventions cannot yet be considered established treatments [119].

Management of thrombocytopenia should reflect bleeding risk and overall clinical condition rather than platelet count alone. Patients without bleeding can often be monitored, whereas platelet transfusion may be considered in selected patients with clinically significant hemorrhage. ZIKV-associated immune thrombocytopenia has been treated with glucocorticoids or intravenous immunoglobulin in individual cases [120]. Supportive care also remains central to severe WNV infection because no virus-specific therapy has been established.

Therapeutic studies should separate virological from clinical endpoints. Entry and replication inhibitors can be assessed by viral load and duration of viremia. Host-directed therapies require inflammatory, vascular, hematological, and organ-specific outcomes. A reduction in ADE of infection does not by itself demonstrate prevention of ADE of disease, just as improvement in inflammation does not necessarily indicate suppression of viral replication.

## 6. Conclusions

Antibody-dependent enhancement remains a major challenge to the development of safe vaccines and antibody-based therapeutics against flavivirus infections. Current evidence indicates that ADE of infection is not determined by antibody concentration or neutralizing activity alone, but arises from the interplay among antibody isotypes and Fc characteristics, activating and inhibitory Fc receptor signaling, host genetic variation, and complement activation. When increased infection is accompanied by Fc-mediated inflammatory signaling and immunopathology, it may contribute to ADE of disease. This distinction has important translational implications: vaccine and therapeutic development should aim not only to elicit potent neutralization but also to minimize enhancing Fc-mediated responses. Rational antigen selection and Fc engineering therefore represent promising approaches to reducing ADE risk. Future studies should prioritize standardized experimental models, prospective clinical validation, and multidimensional immune profiling to clarify the relationship between ADE of infection and ADE of disease. Integrating these data through systems serology and computational approaches may support more reliable risk prediction and the development of safer, more individualized flavivirus interventions.

## Figures and Tables

**Table 1:** Expression, signaling properties, and reported roles of human Fc receptors in flavivirus ADE.

Human Fc Receptors	Principal Ligand	Cell Distribution	Signaling Motifs	Proposed Mechanism and Effect on ADE Reported Relationship with ADE
FcγRI (CD64)	IgG	Monocytes, Macrophages, Neutrophils, Mast cells	ITAM (via the γ chain)	Uptake of virus-IgG immune complexes and activation of downstream inflammatory signaling; promotes ADE of infection and may contribute to ADE of disease [15,16]
FcγRIIa (CD32a)	IgG	Monocytes, Macrophages, Neutrophils, DCs, Basophils, Mast cells, Eosinophils, Platelets	ITAM (via the α chain)	Internalization of virus-IgG complexes, increased infection of permissive cells, and induction of FcγR-dependent signaling; promotes ADE of infection and may contribute to ADE of disease [15,16]
FcγRIIb (CD32b)	IgG	Monocytes, Macrophages, B cells, DCs, Basophils, Neutrophils	ITIM (via the α chain)	Co-ligation with activating FcγRs recruits inhibitory phosphatases (SHIP) and limits immune-complex uptake and cellular activation; inhibits FcγR-mediated ADE of infection and inflammatory signaling [16,17]
FcγRIIc (CD32c)	IgG	NK cells	ITAM (via the α chain)	May promote immune-complex signaling, but flavivirus-specific evidence for a direct role in ADE is limited [16]
FcγRIIIa (CD16a)	IgG	Monocytes, Macrophages, NK cells	ITIM (via the γ chain or ζ-chains	Binding and uptake of IgG immune complexes, macrophage activation, NK-cell activation, and cytokine production; promotes ADE of infection in experimental models and may contribute to disease-associated inflammation [17,18]
FcγRIIIb (CD16b)	IgG	Neutrophils, Basophils	GPI-anchored (no independent signal)	May regulate immune-complex capture and cooperate with other receptors, but direct evidence for ADE is insufficient [16,17]
FcµR	IgM	B cells, T cells, NK cells	None	IgM neutralization, steric competition with enhancing IgG, and complement-dependent viral clearance; no evidence that it directly mediates ADE of infection [19,20]
FcαR	IgA	Monocytes, Macrophages, Neutrophils, Eosinophils, DCs	ITAM (via the γ chain)	Competition with IgG for viral epitopes and immune-complex handling without efficient classical FcγR-mediated viral uptake; IgA has been reported to inhibit IgG-mediated ADE of infection [11,21,22]
FcεRI	IgE	Monocytes, Macrophages, Basophils, Mast cells, DCs, Eosinophils, Platelets	ITAM (via the γ chain)	Mast-cell and basophil degranulation, release of vasoactive mediators, and increased vascular permeability; direct involvement in ADE of infection is unproven, but may contribute indirectly to ADE of disease [23,24]
FcεRII	IgE	Monocytes, Macrophages, B cells, DCs, Eosinophils, platelets	None	May regulate IgE responses and immune-complex handling, but flavivirus- specific evidence for ADE is insufficient [25]
FcRn	IgG	Various cell types	None	Transplacental transfer and prolonged persistence of maternal IgG can produce sub-neutralizing antibody concentrations that subsequently permit ADE through classical FcγRs; does not directly mediate ADE of infection [26,27,28]

Cell distribution refers to human cells. ADE of infection denotes antibody-associated increases in viral entry, cellular infection, replication, or viral load. ADE of disease denotes antibody-associated worsening of clinical or pathological outcomes, including Fc receptor-mediated inflammatory responses. Evidence of ADE of infection in cell-based assays does not by itself demonstrate ADE of disease in humans.

**Table 2:** Summary of Fc region modifications and their effects on ADE of infection and/or ADE of disease.

Modification Type	Target Virus	Effect on Fc Receptor Binding	Reported Effect on ADE of Infection and/or Disease
Afucosylation	DENV	Enhances binding to FcγRIIIa	Promotes ADE [41,42]
Plant-specific xylose and α1,3-fucose in N-glycosylation	DENV, ZIKV	Not clearly defined	Completely blocks DENV ADE and attenuates ZIKV ADE [45,46]
N297A mutation (loss of N-glycosylation)	DENV	Impairs binding to FcγR and C1q	Inhibits ADE [47]
LALA mutation (L234A/L235A)	DENV, ZIKV	Inhibits binding to FcγRIIa and other FcγRs	Inhibits ADE [47,48]
Deletion of aa 231–239 (N-terminus of Fc)	DENV	Abolishes Fc-FcγR binding	Completely eliminates ADE [49]
D265A mutation	DENV	Reduces FcγR binding	Inhibits ADE [47]

## Data Availability

No new data were created or analyzed in this study. Data sharing is not applicable to this article.
